# Exploring the experience of an enhanced recovery programme for gynaecological cancer patients: a qualitative study

**DOI:** 10.1186/2047-0525-3-2

**Published:** 2014-04-04

**Authors:** Stephanie Archer, Jane Montague, Anish Bali

**Affiliations:** 1 Psychology Department, Faculty of Education, Health & Science, University of Derby, Kedleston Road, Derby DE22 1GB, UK; 2 Centre for Patient Safety and Service Quality, Imperial College London, Medical School Building, St Mary’s Campus, Norfolk Place, London W2 1PG, UK; 3 Gynaecology/Oncology, Maternity and Gynaecology Level 2, Women and Children’s Services, Royal Derby Hospital, Uttoxeter Road, Derby DE22 3NE, UK

**Keywords:** Enhanced recovery programmes, Gynaecological cancer, Patient experience

## Abstract

**Background:**

Perioperative enhanced recovery programmes (ERPs), identified as initiatives that improve care and save money, have been adopted by NHS Improvement and are currently being rolled out across many surgical departments within the NHS. To date, five papers have specifically explored patients’ experiences of ERPs; none, however, has explored the gynaecological cancer patient experience.

**Methods:**

In total, 14 women (mean age, 66 years) participated in an audio-recorded face-to-face or telephone interview in which they discussed their experience of taking part in an ERP. The resulting data were transcribed verbatim and analysed using interpretative phenomenological analysis.

**Results:**

Two main themes emerged from the analysis. The first, ‘Taking part in the programme’, highlights two important aspects of the ERP: being given an opportunity to receive information and, following this, to build knowledge about the programme. The theme also explores the challenges associated with the programme, particularly around getting mobile and complying with its demands - the women report experiencing a constant battle between intuition and instruction. The second theme, ‘Home’, focuses on the role home plays in motivating the patients to aim for an early discharge from hospital. Patients describe their need to return to a suitable home and the need for support from others. They also discuss the importance of the follow-up phone call.

**Conclusion:**

Overall, the patients in this study positively assessed the individual aspects of the ERP, in particular, information resources, the availability of the physiotherapist and the delivery of follow-up phone calls. These findings highlight the importance of developing and maintaining individual aspects of ERPs over time, to ensure their sensitivity and responsiveness to patient needs.

## Background

Initiatives such as Lord Darzi’s High Quality Care for All [1], the Strategy for Cancer: Improving Outcomes [2] and the Quality, Innovation, Productivity and Prevention Challenge [3] have all highlighted the need for the NHS to deliver more services but without compromising quality. Enhanced recovery programmes (ERPs) have been identified as initiatives that are beneficial both for improving care and for saving money; as a result these have been implemented in hospital trusts across the country [4]. The aim of ERPs is to ‘improve the quality of patients’ care, through improving their clinical outcomes and experience, and to reduce the length of elective care inpatient pathways across the NHS by utilising good practice principles of the enhanced recovery model of care’ [5].

‘Enhanced’ or ‘fast track’ recovery combines several perioperative interventions to reduce the length of inpatient stay in hospital and to promote early recovery after surgery [6]. Other benefits of ERPs are increased utilisation of beds (that is, available beds having a higher turnover of patients) and reduced healthcare expenditure [7]. The fundamental aspects of ERPs focus on pain control, reduction of surgical stress, enhanced nutrition, heat loss and mobilisation as key factors in patient recovery [8]. These aspects are just a selection of those that have been built into ERPs over the past 15 years [9] and, although originally developed for colorectal surgery, ERPs are now widely used for surgical procedures in both cancer and non-cancer populations.

There are currently only five papers that specifically explore patients’ experiences of ERP; three papers looking at patients with colorectal cancer, and two papers exploring patients’ experiences of hysterectomy [10-14]. Research by Wagner et al. [13,14], explored the patient experiences of ERPs in terms of hysterectomy, although this purposely left out the gynaecological cancer patients ‘because the supplementary diagnosis can have an impact on the women’s experiences of the admission to hospital’ [14]. It is important, therefore, to look at the ERP experiences of cancer patients outside of the colorectal discipline to ascertain the programme's efficacy.

The study reported here explores the experience of a group of women taking part in an ERP at an East Midlands regional cancer centre while having surgery for gynaecological cancer. This qualitative study gives insight into the lived experience of an ERP for this group of women. It provides an in-depth, and previously unexplored, view of the ERP from the gynaecological cancer patients’ perspective.

## Methods

### Participant recruitment

Participants recruited for the study had taken part in the ERP between October 2010 and April 2011 and had undergone elective open surgery for gynaecological cancer. The clinical team identified qualifying patients through hospital records and contact details supplied by the ERP project lead. In total, 34 patients were identified with 33 of these being asked to take part in the study. The 34th patient had returned home to a country outside of the UK and her contact details were not available.

Patients were sent an information sheet, an invitation to participate and a reply slip on which they could indicate whether they were consenting to take part in the evaluation. Those who agreed to take part could choose a face-to-face or a telephone interview. In total, 25 women responded to the invitation; 11 of the respondents opted not to take part in the evaluation. Of the 14 who agreed, four patients chose to be interviewed by telephone and 10 chose face-to-face interviews.

### Participant characteristics and demographics

All patients taking part in the evaluation had undergone a full open hysterectomy, +/- bilateral salpingo-oopherectomy, +/- omentectomy, +/- pelvic lymph node dissection at an East Midlands regional cancer centre. Patients were all living in the East Midlands at the time of the operation and spoke fluent English; the sample comprised of women from various ethnic origins. The age range of the patients was 53 to 80 years (mean age, 66 years).

### Data collection

A semi-structured interview method was utilised, allowing for all participants to be asked similar questions within a flexible framework [15,16]. All interviews were conducted in a place suitable for the women (which was chosen by them) and were recorded on a digital voice recorder. Participants dictated the flow of the interview, covering topics in an order with which they felt most comfortable. Questions focused on the women’s experiences of participating in the ERP as well as any other aspects of their hospital care they felt to be important. If further clarification or exploration of a topic was required, the researcher ‘revisited’ these topics before the close of the interview.

The study conformed to British Psychological Society ethical standards [17]; the protocol was approved by the University of Derby Psychology Ethics Committee and was approved as a current audit by the NHS hospital audit team at the Royal Derby Hospital. Participants gave written informed consent, and pseudonyms were used to protect their anonymity. Patients were also debriefed after their participation in the study and directed to suitable support agencies where necessary.

### Analytic procedure

The choice of a qualitative methodology for this study was informed by researchers such as Starks & Brown Trinidad [18], who state that ‘qualitative research methods enable health sciences researchers to delve into questions of meaning, examine institutional and social practices and processes, identify barriers and facilitators to change and discover the reasons for the success or failure of interventions’. Analysis was conducted using an interpretative phenomenological analysis (IPA) approach, a methodology frequently used in health services research [15,19-22]. IPA was developed to ‘explore the participant’s view of the world’ and to adopt, as far as possible, an ‘insider perspective’ [23] and is informed by phenomenology and hermeneutics [24].

In line with the IPA methodology, interviews were transcribed verbatim, and initially coded by reading and re-reading the transcript and making notes, drawing on observations made during the interviews and transcription. Transcripts were then coded line by line: describing, summarising and attending to linguistic elements such as pronoun and metaphor use [15]. Emergent themes were developed from these codes and clustered with related themes.

## Results

Two emergent themes are explored in this paper: ‘Taking part in the programme’ and ‘Home’, both of which focus on the role of ERP in the medical setting. The theme of home is further split into two subthemes: ‘Going home’ and ‘The follow-up phone call’.

### Taking part in the programme

Overall, the introductory information provided to these women about enhanced recovery was reported as informative both for them and for significant others. Patients described available information as increasing their knowledge; leading to a greater understanding of why they were being asked to comply with each part of the programme.

“So you know everything went so smoothly and when he said you are going to go into that fast-track programme, I thought well that’s absolutely splendid… I was absolutely thrilled to bits, you know, and I must admit I had all the details, the information that was given to me was terrific, I felt perfectly confident and happy about everything… and I took it home anyhow and studied it, because I thought if I am fast-tracking I want to be part of this” (Betty 42-88).

Existing health psychology literature recognises that seeking and using information (or not) can be indicative of, or linked to, coping style [25-27]. Those patients who use a problem focused coping strategy are suggested to benefit from the ERP’s enhanced information as it helps them to understand their experience [28-30]. This was reflected in this group’s reported experiences, which suggested that they were more likely to use problem focused than emotion focused coping strategies in dealing with their illness. Enabling patients to actively participate in their own care through being informed maintains a sense of autonomy which in turn prompts their motivation to adhere to a care programme [31].

Patients cited getting active as an important part of the programme, and receiving information and gaining knowledge enabled them to achieve this more quickly. It seemed that knowing what was expected of them and why helped them regain the control that is often reported as lost when undergoing surgery [32,33]. For this group, being active was the key to going home; once this was achieved then their return home was assured. The route to being active, however, was not straightforward and a number of barriers were identified, such as getting out of bed:

“The fact that I knew what I was going to have to do when I came round. I knew I was going to have to get up, and I knew that I had to get up and walk. The preparation is good” (Lynn 564-567).

In some ways the ERP, with its emphasis on getting up and active rather than resting and recuperating, contradicts the traditional post-surgery behaviour that this group of women would be more familiar with. A number of them had undergone gynaecological surgery in the past and most had had contact with someone else who had undergone a similar experience; being asked to get up almost immediately seemed, therefore, to be counterintuitive.

“I think there is a fright in moving because, well I had a big scar that does right across my abdomen… but it was this horrible feeling that you are going to burst it or something, because it’s always there I suppose in your mind that are the clips going to hold? But you know, you have to have confidence in all of these things. They have done it thousands of times before, so it’s very rare that you would have a big problem as long as you do, you move how they tell you” (Lynn 205-216).

Although patients included in an ERP are told that they will be expected to get out of bed, this does not transfer into intention, and subsequently, behaviour. There are several reasons why patients do not want to get out of bed and mobilise, such as discomfort or pain or because they believe that they cannot, or even that they should not, be getting out of bed so soon.

“I think there were about three things and I was doing them all the time, to keep everything going. I knew I had got to get off the bed as much as I could, but you really don’t want to. You don’t want to” (Sharon 347-349).

The role of the physiotherapist is integral to getting patients out of bed; they successfully get patients out of bed and encourage them to be mobile [34,35]. Postoperatively, in particular, the visit from the physiotherapist forms a key part of the start of the mobility aspect of the programme. By giving the patient permission to mobilise, the physiotherapist can build confidence that no harm will result.

“Well I think they took the, the drip thing out and then the physios were round quite early, and they got me out of bed, and then I’d still got the catheter in - that was the thing that bothered me the most before I went in, was having a catheter… so the physios came round and sort of helped me out of bed, and made sure I did it the right way. But I’d been practising at home, but it’s not quite the same is it when you get there. And she took me for a walk along the corridor, and went through things again, what I should be doing” (Jane 418-430).

Once they are out of bed, patients report that being mobile is not as difficult as they expected. This enables them to move towards completing ‘everyday’ tasks such as getting dressed, brushing their teeth and bathing. This return to ‘normal activities’ is important post-surgery; it helps patients take some control of their own care and builds confidence. The women in the current study reported a belief that these are the first steps on the road to being able to return home and their rapid resumption of physical activity suggests a much less stressful post-surgery experience than might usually be the case.

### Home

#### *Going home*

On Day Two of the ERP, patients are up and out of bed, can eat normally, have been to the toilet and have had a shower; they feel that they are ready to go home and they believe that this is possible. Patients in this sample felt that they had completed their ‘end of the bargain’ so should be given the reward of going home. Home seems to be the desirable place for discharge for most patients and all patients in this sample were being discharged to their own home. Previous research highlights that patients on ERPs who are not discharged to their home (so to a nursing home or to hospital) do not have as reduced a length of stay as those who are. It seems, then, that a return to ‘own home’ is a motivator for early discharge [36].

“Yes, yes definitely, because I knew that I would be going home quicker and you feel better at home, you feel more relaxed at home, and I just felt better going home, sooner be at home” (Liz 310-313).

Similarly, recovery is discussed as being easier to achieve in more familiar surroundings.

“The all round business of being able to get and move more easily at home, I mean there is no doubt that I began to recover the minute I got home” (Betty 368-370).

It appears, then, that in terms of this sample, home has a special significance which seems to be more than just ‘not wanting to be in hospital’. Home is personal and symbolises a certain level of normality in their lives, especially after diagnosis. This desire to return home more quickly was not as apparent in all cases, however, and if the patient did not feel fully recovered, home was a less attractive option.

“No, I was panicking at that point because I felt so rough when I woke up on the Friday morning and I said to him [consultant], “well I don’t know if I want to [go home] because I didn’t feel well this morning”, and he said “well I’m happy, I know you are going to be cared for at home, quite well, I’ve met your husband, I know you will be cared for. If you’re up to it, you can go home…” and I said “can I stay because I really don’t feel too good”” (Sharon 455- 460).

Overall, then, this group of patients report a lack of pleasure in being in the hospital environment when they feel ‘well’ and express a strong desire to go home. On the other hand, they feel that the hospital environment is beneficial for their recovery, when they feel that they are not yet recovered. This may be because they do not want to leave hospital when they are unwell, or because they do not want to return to the home environment feeling ill.

Home is related to normality and the illness (in this case surgery for cancer) has disrupted that normality for them. After surgery, familiar activities become more difficult. Home, therefore, can be viewed as restrictive: what was once easily performed in a familiar environment is now difficult (for example, climbing the stairs). Traditionally, hospital has allowed patients to experience those negative aspects of illness (in this case recovery from surgery) in an external environment, thus leaving the home intact and devoid of the experience of illness. This idea is challenged by ERPs: patients return home earlier in their recovery journey, while everyday tasks are still difficult, thus blurring the boundaries between hospital and home. The early return home also necessitates a different level of support, at least for a while.

“I had our mum in law, my mum in law live with me for a while so we had got a stair lift, so that was very handy because I would have never took the stairs because we are in an Edwardian house with very steep stairways, so I wouldn’t have got up and down” (Lily 311-316).

Previous research has found that having a partner was positively associated with engagement in activities such as reading, washing oneself, ambulating and exercising in the days following surgery [37]. The analysis reported here reveals that significant others (generally husbands in this sample) are required to fulfil a number of functions including carer, enforcer of rules and companion. In some circumstances this marks a pronounced change or reversal of roles within the household with significant others receiving little or no preparation regarding their involvement in, and the practicalities associated with, having to care for someone in the early stages of recovery.

“And the consultant explained again that if everything was OK he’d check again on the circumstances at home, and that James would be at home for a while, he said that if he was happy to have me home, then there would be no reason why I couldn’t go home” (Sheila 111-116).

Patients often feel that they can do more than they are ‘supposed to’ resulting in partners or family members policing them to prevent them from doing things that they should not.

“I know they say no lifting for 6 weeks afterwards, you could almost, I almost felt as if I could get, if I didn’t have my husband around saying don’t do that, I’ll lift that and all the rest of it, I did feel extremely well” (Rachel 276-287).

The idea, from this sample, that they can do more than asked is reinforced by their completion of the tasks set by the hospital and the consequent reward of discharge. Once discharged, though, patients struggle to reconcile intuition and instruction; they generally feel well and are able to attempt or complete many ‘everyday’ tasks around the home, without always needing the support from their significant other. They become the ‘recovering patients’ in comparison to the ‘active patients’ that they have been in hospital. Furthermore, they are not just ‘recovering from the operation’ but are moving to ‘recovering from cancer’.

#### The follow-up telephone call

In the role of ‘recovering patient’ at home following the ERP it is important to maintain and continue communication with the hospital. The analysis conducted for this study revealed, however, that there was a clear breakdown of communication once these patients returned home. Many feel as if they are alone and are reluctant to call for assistance from the hospital even if it is to ask for advice. ERP patients are instructed to call the hospital if they have any questions or queries once they get home. This avenue of communication is one of the reasons that ERPs work: though the care is transferred from the hospital to the home this communication channel eases the transition from one to the other [5]. The current analysis suggests otherwise: little communication is reported and patients feel uncomfortable contacting the hospital, even if there is a problem.

“Yes, yeah, because I think that even though they say that if you’ve got any problems you can ring us, well I know, I don’t know other people but, but me personally you know, I, I know that I tend to leave things a bit too long maybe, and I don’t like to bother people, and I probably wouldn’t have phoned unless I was really, really worried. (Jane 573-580).

The project lead for the ERP explored here initiated the use of follow-up phone calls to ease the transition from one environment to the other. This initiative met with some success and is supported by research conducted in other areas [38,39]. The analysis clearly highlights that patients value the follow-up phone call and believe that this is beneficial in their transition to being at home.

“It was nice to know that she was going to ring when I got out of the hospital, because I thought I’ve got the weekend now, and, am I going to be alright, I mean I don’t want to be a nuisance, although the ward had reassured me to ring if there was a problem. But I didn’t want to sort of be a nuisance as such, and I was a bit worried that what would happen just in case they were any problems, but it was nice to know Katy was going to call on Monday” (Sheila 1010-1024).

The use of telecommunication is not new, but is a resource that is becoming increasingly beneficial for those in the medical profession. The ability to be able to contact patients outside of the hospital environment is of benefit to both the patient and the hospital, as it means that valuable bed days are saved for the hospital and that patients are reassured that they are not expected to manage on their own. The follow-up phone call provides an opportunity through which information can be reinforced, which may increase compliance whilst ensuring that patients are both physically and emotionally comfortable [40]. It also means that both sides are given the opportunity to give and receive important information.

“Yes, yes the enhanced recovery people actually phoned up to the ward to see how I was doing, and when I got home… they wanted to see how I was doing. They had pre warned me that they were going to be keeping an eye on me which was nice really” (Lily 328-335).

Of course, one challenge is that the calls *must* happen: patients must have that contact with the hospital if they are expecting it, in the same way as if a visit from a doctor was promised at the hospital; deviation from the expected can lead to a negative experience for patients, as they may well be relying on the follow-up phone call from the hospital to discuss any difficulties or to ask any questions that they may have after discharge. Not implementing the follow-up call may result in other healthcare providers having to see patients in clinic or in the home (GP practices or district nurses) when a follow-up phone call may have dealt with the question in a more timely and efficient manner.

## Discussion

### Main findings

Prior to surgery, the receipt of information about the coming days is particularly important for patients and their significant others. It allows them to understand why they are being asked to comply with the programme and helps set their expectations about what is required from them after surgery. This information moderates the relationship between instruction and intuition, which is an ongoing battle for patients taking part in ERPs. Patients find that initiating physical movement (getting out of bed) most difficult; physiotherapists have an important role to play in patients’ mobility as they, in part, give patients both permission and confidence to mobile within a structured environment.

Patients who see a physiotherapist at the preoperative appointment are shown how to get off the bed, and are given information to support this. This process was highlighted in the patients’ accounts of their experience as being important for them, so is something that should be encouraged, particularly as it may improve patients’ self-efficacy in getting out of bed and starting to mobilise [41-43]. It is known that self-efficacy represents a personal resource factor that may facilitate coping both during and after surgery [44]. Bandura described that one of the ways to improve self-efficacy is though vicarious experience (watching someone else who is comparable with the patient) [43]. In light of this, it may be worthwhile having a resource available for patients to watch or use that shows patients how to correctly get out of bed (modelled by an appropriate actor), in something as simple as a video that could be distributed on CD, online or shown in clinic [45].

ERPs allow patients to go home sooner, and for many this is the place that they desire to be (albeit when they are confident to return home). However, home is not as ‘easy’ as before the operation. Early discharge with reduced mobility alters the role of home and the interaction that patients have with this place. Additionally, early discharge challenges the roles of those who are associated with home (primarily spouses or other close family members). Information about what patients should and should not do after their operation is detailed in the literature given to them at the preoperative stage and is followed by confirmation on discharge. As long ago as 1999, however, researchers found that physicians overestimate patients’ understandings of the post-discharge treatment plan, and suggested that steps should be taken to improve communication about post-discharge treatment [46]. More recently, research has found that husbands who were asked to watch videotaped information designed to assist patients in their recovery after surgery were more successful in helping their wives achieve this, thus suggesting that information specifically written for significant others may be of benefit [47].

The implementation of follow-up phone calls assists with the transition from the hospital to the home environment. The analysis highlights that patients are unlikely to contact the hospital themselves. Therefore, the follow-up phone calls must happen in order to prevent unnecessary readmission and use of primary care resources. An intervention that would encourage patients to call the ward when necessary would be beneficial. This may include some sort of prompt sheet in the discharge material that details the type of problems to look out for (that is, with wounds or bowel movements). This would be beneficial for both patients and significant others as it would raise awareness of some of the issues that are associated with this type of surgery and which ones are problematic and require hospital intervention. In addition to this, it may be beneficial for those completing the discharge to emphasise the availability of contact with the ward to the significant other who is staying with the patient on their return home. The significant other often becomes the main carer and is on occasion faced with the decision about whether to seek medical assistance [11]. The availability of communication with a healthcare professional on the ward may well reduce some of the worry or stress associated with caring for a patient at home who is on day two of recovery from surgery.

### Strengths and limitations

This is the first study looking at the experiences of taking part in an ERP for women undergoing surgery for gynaecological cancer. The analysis of the interview data gives a previously unseen view of ERPs from the patient experience and has highlighted a number of areas for consideration when designing perioperative care for gynaecological cancer patients. However, there are a number of limitations. Patients included in the analysis were recruited from a larger sample of women. All patients on the ERP within a given time frame were asked to take part, and out of 33 patients, only 14 chose to participate. This self-selecting sample may have been the patients who have ‘something to say’ about the programme and chose this evaluation as an avenue for discussion. Further to this, the interviews were conducted after a considerable period of time for some patients (up to 8 months after the operation). For future research it may be beneficial to interview patients closer to the time of surgery. However, for this study it was difficult to interview participants who were still on active treatment, as they did not have time to participate, and in some cases, did not feel ready to explore their experiences of surgery.

This sample also consisted of older women. With a mean age of 66 years, there was little reflection of the breadth of ages of patients with gynaecological cancer. Younger patients were invited to take part but declined the offer. It would be interesting to compare the experience of younger patients (that is, those with cervical cancer) with that of the older generation to explore any impact of age on the perception of the programme and their expectations. In addition to this, all patients in this sample were returning to their own home rather than an alternative place of discharge such as a nursing home or a long-term hospital bed. Research suggests that patients who return to a destination other than home experience longer lengths of stay than those who return home [36]. As the current analysis had such a large focus on home and the role of home in enhanced recovery, additional research with a diverse patient group is required to effectively explore other possible experiences.

## Conclusion

ERPs are highly valued by the patients taking part in them. For patients, ERPs are more than just a reduction in length of stay. Patients value the individual aspects of the ERP, in particular, information resources, the availability of physiotherapists and the delivery of follow-up phone calls. This highlights the need to develop and maintain the individual aspects of ERPs over time to ensure that ongoing developments in the programme are sensitive and responsive to patient needs. Ongoing research into the patient experiences of ERP are required to ensure that quality care is being delivered to patients, and that the patient experience is considered alongside length of stay data in terms of determining the efficacy of ERPs.

## Competing interests

There are no competing interests for any of the authors of this paper.

## Authors’ contributions

SA, JM and AB were all involved in the planning of the research, the analysis of the data and the preparation of the manuscript. All authors approved the final version of the manuscript.
